# A silent network’s resounding success: how mutations of core metabolic genes confer antibiotic resistance

**DOI:** 10.1038/s41392-021-00717-x

**Published:** 2021-08-12

**Authors:** Gamal Wareth, Heinrich Neubauer, Lisa D. Sprague

**Affiliations:** 1 Friedrich-Loeffler-Institut, Institute of Bacterial Infection and Zoonoses, Jena, Germany; 2 Department of Bacteriology, Mycology and Immunology, Faculty of Veterinary Medicine, Benha University, Toukh, Egypt

**Keywords:** Infectious diseases, Metabolic engineering

A largely underrated factor in antibiotic resistance is the role of metabolic mutations. In a recent paper published in *Science*, Lopatkin et al.^1^ used different in vitro evolution protocols and extensive sequencing data analyses to demonstrate that metabolic mutations develop in response to antibiotic treatment in clinically relevant bacteria (Fig. 1).

**Fig. 1:**
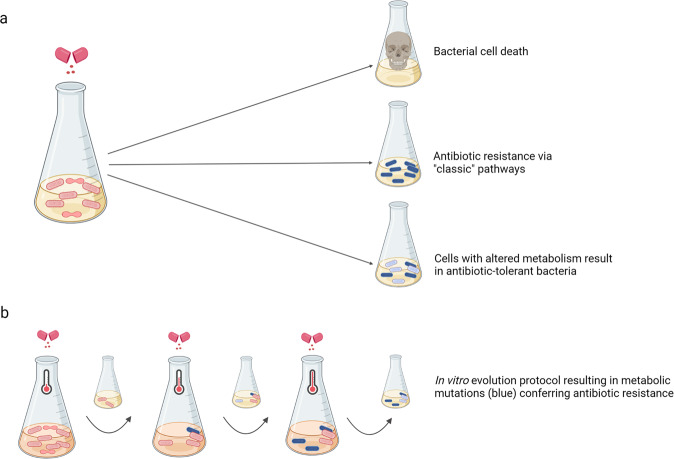
**a** Antibiotic treatment can result in cell death, development of antibiotic resistance through target modification, drug inactivation and drug transport or mutations in core metabolic genes enriching for metabolic specific pathways leading to adaptation. **b** In vitro evolution protocol applied by Lopatkin et al. to generate predominantly metabolic mutations. Cells were subjected to daily 1-h antibiotic treatment and 22 h of growth without antibiotics. Metabolic activity was modified by the daily increase of temperature over a period of 10 days

Bacteria acquire resistance by means of various mechanisms, such as enzymatic inactivation, changes in target- or binding sites, alterations in membrane permeability resulting in increased drug efflux or reduced drug permeability, gene mutations or by horizontal gene transfer. Bacterial metabolism, however, has only recently been acknowledged as a factor conferring antimicrobial resistance, since mutations resulting in antibiotic resistance are only infrequently identified in metabolic genes. As such, a recent study investigating metabolic rearrangements during the evolution of antibiotic resistance in *E. coli* revealed, that carbon and energy metabolism strongly constrained the evolutionary trajectories in terms of speed and resistance acquisition.^2^ But also other studies support the idea that mutations in metabolic networks have an impact on antibiotic efficacy, as antibiotics alter the metabolic state of bacteria, thereby contributing to bacterial death, e.g. via oxidative damage or stasis through translation inhibition resulting in decreased cellular respiration. The bacterial metabolic state in turn, has an influence on antibiotic susceptibility and by altering the metabolic state of bacteria, antibiotic efficacy can be improved.^3^

Mathematical models such as the flux balance analysis (FBA) can simulate metabolism in genome-scale reconstructions of metabolic networks. In a preceding study using FBA, Lopatkin et al. studied the effects of varying concentrations of glucose and casamino acid on a genome-scale model of *E. coli* metabolism.^4^ Based on the obtained data and in vitro experiments, they were able to determine the relative contribution of growth and metabolism to antibiotic lethality across a broad range of coupled and uncoupled conditions. Moreover, they were able to show that antibiotic lethality depends on the bacterial metabolic state at the time of treatment, rather than the growth rate. These findings applied to numerous conditions, including several representative bactericidal substances as well as a diverse range of Gram-positive and Gram-negative species.^4^

In the presently discussed study, Lopatkin and co-workers, again using *E. coli* as a model pathogen, provided proof that metabolic mutations can develop in response to antibiotic treatment.^1^ This was achieved by applying different in vitro evolution protocols and subsequent sequencing analyses of entire populations and selected clones. In a first step, the authors used a traditional protocol of serial passaging and continuous exposure to increasing concentrations of three representative bactericidal compounds targeting DNA replication (ciprofloxacin), cell wall biosynthesis (carbenicillin) and protein synthesis (streptomycin) to induce growth-dependent selection. The subsequent comparison of the sequence data from entire populations and individual clones revealed that more mutations of metabolic genes were found at the population level than at the clonal level, albeit at low frequency, indicative of an underlying genetic network of mutations with a previously unknown impact on metabolism influencing the evolution of resistance to antibiotics. Lopatkin et al., therefore, drew the conclusion that classical experimental evolution together with the sequencing of candidate genes and/or a small number of clonal isolates per condition mostly reveals expected mutations, or those occurring at a higher frequency but masks those occurring more infrequently. Consequently, in order to increase the frequency of metabolic mutations during the evolution of antibiotic resistance, the authors cycled *E. coli* subjected to daily 1-h antibiotic treatment and 22 h of growth without antibiotics. Metabolic activity was modified by daily increasing the temperature by 1 °C over a period of 10 days starting at 20 °C. This new protocol generated predominantly metabolic mutations in the emerging strains that were solely attributable to the exposure to antibiotics and not to the alternating growth/no growth cycle conditions. In the next step, the authors compared the biological processes significantly affected by the classic and the metabolic evolution protocols and observed the occurrence of parallel metabolic mutations indicative of metabolism modulation as an adaptive strategy against antibiotic stress. In order to assess the clinical relevance of these mutations, Lopatkin et al. analysed a library of 7234 *E. coli* genomes from clinical and non-clinical origin deposited at NCBI using the *gyr*A gene as baseline for comparison. Approximately 20% of the strains from either clinical or non-clinical origin had a mutation in *gyr*A_S83 and 12% in *gyr*A_D87, indicating that mutation prevalence alone can have a potential clinical impact. In order to test if the found metabolic mutations conferred resistance, the authors then evaluated a representative subset of metabolism-related genes (*suc*A, *glt*D, *ush*A, *icd*, *ycg*G and *yid*A) and classic antibiotic resistance genes (*omp*F, *acr*D and *gyr*A). Knockout and overexpression experiments, as well as measurement of growth inhibition confirmed that metabolic mutations indeed lead to an increase of the minimum inhibitory concentration to at least one antibiotic in all cases, and in some to more than one. Finally, by analysing a mutation in the 2-oxoglutarate dehydrogenase enzyme (*suc*A), the authors were able to show that metabolic mutations per se can result in a small but measurable increase in antibiotic resistance.

The work of Lopatkin et al. has shed light on the intricacies of metabolic mutations and their direct influence on antibiotic resistance and has provided a fresh outlook on the development of antibiotic resistance.^1^ Their findings could help to explain the occurrence of (multi-) drug-resistant bacterial strains isolated in regions without significant exposure to antibiotics or the observed increase in antibiotic resistance upon excessive application of herbicides or other environmentally toxic substances.^5^ Systematic monitoring and analysis of the metabolic response to bactericidal as well as bacteriostatic compounds can provide a means to optimise and possibly even personalize treatment regimens and to test the efficacy of new drugs or new drug combinations of known compounds. In view of the global rise of antibiotic resistance not only expected to cause millions of deaths in the next decades but also putting significant pressure on national economies, new treatment options are in dire need. Lopatkin et al.’s work has come at exactly the right time.

## Supplementary information

Publication and Licensing Rights
