# Supplementary material for: A silent network’s resounding success: how mutations of core metabolic genes confer antibiotic resistance
Source: Signal Transduct Target Ther. 2021 Aug 12;6:301. doi: 10.1038/s41392-021-00717-x (PMC8361174; doi:10.1038/s41392-021-00717-x)
Supplement: Supplementary file 1 — Publication and Licensing Rights [file 41392_2021_717_MOESM1_ESM.pdf]

639 Queen St. W Suite 401
Toronto ON M5V 2B7 Canada
www.biorender.com

Confirmation of Publication and Licensing Rights

July 2nd, 2021
Science Suite Inc.

Subscription: *Individual*
Agreement number: *NS22NTPC86*

To whom this may concern,

This document is to confirm that **Lisa Sprague** has been granted a license to use the BioRender content, including icons, templates and other original artwork, appearing in the attached completed graphic pursuant to BioRender's Academic License Terms. This license permits BioRender content to be sublicensed for use in journal publications.

All rights and ownership of BioRender content are reserved by BioRender. All completed graphics must be accompanied by the following citation: "Created with BioRender.com".

BioRender content included in the completed graphic is not licensed for any commercial uses beyond publication in a journal. For any commercial use of this figure, users may, if allowed, recreate it in BioRender under an Industry BioRender Plan.

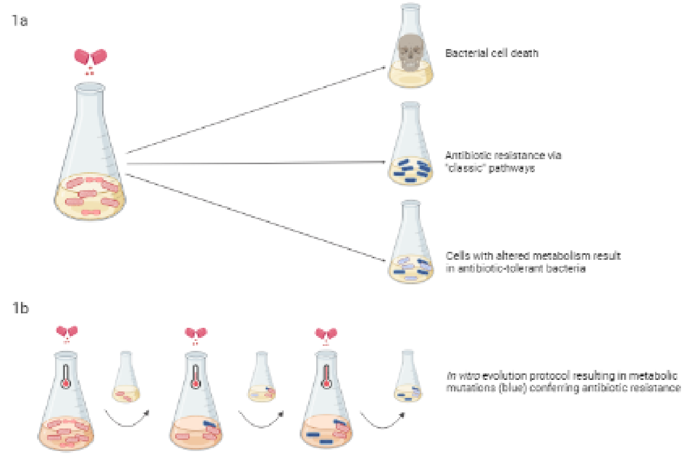

Figure 1
a. Antibiotic treatment can result in cell death, development of antibiotic resistance through target modification, drug inactivation and drug transport or mutations in core metabolic genes enriching for metabolic specific pathways leading to adaptation.
b. In vitro evolution protocol applied by Lopatkin et al. to generate predominantly metabolic mutations. Cells were subjected to daily 1 -hour antibiotic treatment and 22 hours of growth without antibiotic. Metabolic activity was modified by daily increase of temperature over a period of 10 days.

For any questions regarding this document, or other questions about publishing with BioRender refer to our BioRender Publication Guide, or contact BioRender Support at support@biorender.com.
